# Waterpipe Smoking among University Students in Sulaimaniyah, Iraqi Kurdistan: Prevalence, Attitudes, and Associated Factors

**Published:** 2017

**Authors:** Nasih Othman, Attallah O. Kasem, Faisal A. Salih

**Affiliations:** 1 Kurdistan Institution for Strategic Studies and Scientific Research, Sulaimaniyah, Iraq; 2 Department of Community Health, Sulaimani Technical Institute, Sulaimani Polytechnic University, Sulaimaniyah, Iraq; 3 Department of Medical Laboratory, Technical College of Health, Sulaimani Polytechnic University, Sulaimaniyah, Iraq

**Keywords:** Waterpipe smoking, Iraq, Youth, Risk factors

## Abstract

**Background:**

Waterpipe smoking is increasingly becoming the most common method of tobacco use among adolescents in the Eastern Mediterranean Region. This study was undertaken in Iraqi Kurdistan to estimate its prevalence among students and investigate attitudes and factors associated with it.

**Materials and Methods:**

In a cross-sectional survey at Sulaimani Polytechnic University, 1160 students were approached in a two-stage design using a self-administered questionnaire. Data was entered into Epidata and analysis was done in Stata.

**Results:**

Prevalence of cigarette smoking was 10% and waterpipe smoking was 28% (male 49%, female 10%). Waterpipe smoking was initiated prior to joining the university in 74% of the cases and 22% of waterpipe smokers smoked every day. The most common place for smoking was coffee shops (52%) and 71% of smokers shared the pipe. The significant risk factors were smoking cigarettes (OR 10.3, 95% CI 7.0–15.0), male gender (OR 5.7, 95% CI 3.9–8.2), non-Kurdish ethnicity (OR 3.0, 95% CI 1.6–15.9), city residence (OR 1.5, 95% CI 1.0–2.1), and use of alcohol and other substances (OR 2.8 95% CI 1.4–5.6).

**Conclusion:**

Waterpipe smoking is highly prevalent among students in Iraqi Kurdistan, especially among males, and is becoming a public health problem. Tobacco control interventions should be designed specifically to address this problem among adolescents and the youth.

## INTRODUCTION

According to the WHO, there are over 1 billion smokers worldwide with a global prevalence of 21% among adults in 2013(1). Waterpipe smoking (WPS) has increasingly become a common method of tobacco use worldwide, and it is the most common method of tobacco use among adolescents in the Eastern Mediterranean Region (EMR) according to Maziak et al. study (2). A number of studies have been undertaken in several countries of the region to estimate the prevalence of WPS and associated attitudes especially among adolescents and the youth and have reported varying prevalence rates. A study in Oman reported a prevalence rate of 9.6% among adolescents in 2008(3), while another from Syria reported 23.5% among university students in the same year(4). Studies in other countries have reported similar or higher rates such as 6% among adolescents(5) and 51% among university students in Iran(6), 29.5% among students in Lebanon(7), and 30% among students in Jordan (8). It can be noted from the literature that the prevalence of WPS has been increasing in the EMR countries. Although WPS was traditionally present in Iraq for the more affluent people, coffee shops with WPS facilities have become commonplace in recent years and widely available to the youth. Despite the extent of the emerging problem and its potential health implications for smokers, the problem has not yet received necessary attention from health authorities and researchers. Currently, there are no published studies on waterpipe smoking in the Iraqi Kurdistan region. Therefore, the current study was undertaken to address this gap and estimate its prevalence among university students and investigate associated attitudes and factors. This information could be useful for designing public health interventions and further research to address the health issue.

## MATERIALS AND METHODS

A cross-sectional survey was undertaken at Sulaimani Polytechnic University’s 10 campuses located in different towns of Sulaimaniyah province, Iraqi Kurdistan. This university enrolls around 13000 students at its 8 institutes (2 years of education) and 5 colleges (4 years of education). Sample size calculation was done in EpiInfo version 7.0 using the following parameters: reference population of 7110 students (1^st^ year students were not included in the study), estimated WPS prevalence of 10%, 2% error level, and a design effect of 1.5. This calculation gave a sample of 1160 students. Sampling was done in three stages. At the first stage, the sample was divided proportionate to population (student) size among colleges and institutes; at the second stage the sample of each college/institution was divided proportionate to population size by gender; and at the third stage individual students were selected using simple random sampling. A self-administered questionnaire was developed in the local language based on a review of previous studies(6, 9, 10). The questionnaire was shared with experts to ensure face validity, revised, and then piloted with a sample of students to make sure it was valid, reliable, acceptable, and accurately understood. The questionnaire included variables on sociodemographic characteristics (age, gender, residence, and ethnicity), cigarette and waterpipe smoking habits, and attitudes about waterpipe smoking. Ethical principles were followed. After obtaining informed consent from participants, they were requested to complete the questionnaire. At the beginning of the questionnaire, a statement was included to inform participants about privacy, confidentiality, and voluntary participation. Data were collected between December 2014 and February 2015. A waterpipe smoker was defined as a person smoking a waterpipe at least once a month. Cigarette smokers were divided into regular smokers (currently smoking every day) and occasional smokers (including people who do not consider themselves smokers and only smoke rarely on certain occasions). Data were entered into EpiData version 3.1(11) and analysis was done in Stata version 13.0(12) using the “Survey Data Analysis” option that accounts for the design effect in reporting weighted estimates. Frequencies of attitudes and beliefs about WPS were analyzed for the entire sample followed by comparing males and females using a chi-square test. To investigate risk factors of WPS, waterpipe smokers and non-waterpipe smokers were compared using a chi-square test. Factors found significant at the 0.2 level were included in the multivariate logistic regression model. P values were reported as calculated in Stata but P values smaller than 0.001 are reported as <0.001. Strobe checklist for reporting cross-sectional studies(13) was followed in reporting the study.

## RESULTS

The calculated sample was 1160 students of which 1061 students returned the questionnaire, giving a response rate of 91%. The response rate was 93% for males, 90% for females, 95% for college students, and 90% for students from the institutes. Respondents included 817 (77%) students from institutes and 244 (23%) students from colleges. The weighted percentages for different characteristics of these students are shown in Table 1. Females were 53% of the respondents. The majority of the respondents were in their second year of education (86.5%), which includes all participants from institutes given the sampling excluded first year students. Over 89% of the respondents were residents of Sulaimaniyah Province of which 36% were from the city center and the remainder were from other provinces. Of all participants, 298 reported WPS amounting to a prevalence of 28% (males 49.4%, females 9.4%). Prevalence of regular cigarette smoking among students was 10%. A total of 10% of respondents reported use of alcohol and other substances. Reported parental cigarette smoking was 23%.

**Table 1.** Main characteristics of the sample

**Characteristics**	**Number**	**Per cent**
All	1061	100
**Sex**		
Female	565	53.0
Male	496	47.0
**Education**		
College	244	23.0
Institute	817	77.0
**Stage**		
Second year	914	86.5
Third year	89	8.1
Fourth year	58	5.4
**Residence**		
Sulaimani city	372	35.6
Sulaimani Province	549	53.8
Other provinces	115	10.6
**Ethnicity**		
Kurdish	1051	99.2
Other*	8	0.8
**Waterpipe smoking**		
Yes	298	28.0
No	763	72.0
**Occasional cigarette smoker**		
Yes	217	21.4
No	795	78.6
**Regular cigarette smoker**		
Yes	100	9.9
No	913	90.1
**Parents smoke cigarettes**		
Yes	230	22.6
No	801	77.4
**Alcohol & other substances**		
Yes	74	7.5
No	943	92.5
Mean age in years(SD)	21.7 (0.08)	

* Includes Arabs, Turkmen and others

### Characteristics of the waterpipe smokers

Table 2 shows various characteristics of the waterpipe smokers. The mean age of waterpipe smokers was 22.0 years (*SD* = 2.4), but the majority of waterpipe smokers (74%) started smoking a waterpipe before joining the university, i.e., before 18 years of age. The duration of WPS was one year or more in 76% of smokers. A total of 22% of waterpipe smokers smoked every day. The most common place for waterpipe smoking was coffee shops (52%) and 71% of smokers shared the pipe. While 52% of smokers reported their intention to quit the behavior, 49% had tried to quit it in the past.

**Table 2.** Characteristics of the waterpipe smokers

**Characteristics**	**Number**	**Per cent**
All	298	100
**Waterpipe history**		
Started before admission to university	215*	74.2
Started after admission to university	75	25.8
**Duration of waterpipe smoking**		
Less than one year	65	21.6
One year or more	222	76.4
**Frequency of waterpipe smoking**		
Every day	60	22.1
At least once a week	78	28.7
At least once a month	134	49.2
**Parents aware of the behaviour**		
Yes	156	53.3
No	136	46.7
**Siblings aware of the behaviour**		
Yes	194	65.9
No	98	34.1
**Share waterpipe with students**		
Yes	205	70.9
No	81	29.1
**Waterpipe smoking place**		
Café	145	51.7
Home	68	23.2
Friends’ homes	10	3.2
Student hostels	19	6.6
Other places	44	15.3
**Intent to quit**		
Yes	149	52.4
No	138	47.6
**Quit attempt before**		
Yes	141	49.2
No	146	50.8

* The numbers may not add up to 298 because of missing values

### Attitudes and beliefs about waterpipe smoking

Table 3 shows knowledge and attitudes of all participants about waterpipe smoking. Almost 67% of the participants said that waterpipe smoking was more harmful to health than cigarette smoking, and 33% said it was socially more acceptable than cigarettes. When nonwaterpipe smokers were asked whether they intended to start WPS, only 1.6% said that they intend to. Comparing waterpipe smokers and non-waterpipe smokers in relation to these attitudes showed significant differences as shown in Table 3. For example, while 89% of non-waterpipe smokers believed waterpipe smoking may cause addiction, only 62% of smokers believed so, and 70% of non-smokers vs. 57% of smokers believed that waterpipe smoking is more harmful to health than cigarettes. A total of 71% of smokers and only 8% of non-smokers said waterpipe smoking is “cool”; 63% of smokers and only 37% of non-smokers believed that waterpipe smokers have more friends. See the table for other comparisons.

**Table 3.** Comparison of waterpipe smokers and non-waterpipe smoker in relation to knowledge and attitudes about WPS

	**All (n=1061)**	**Waterpipe smokers (n=298)**	**Non-waterpipe smokers (n=763)**	**P value (Design-based)**

	**Number (%)**	**Number (%)**	**Number (%)**	
**Waterpipe smoking may causes addiction**	832(81.7)	177(62.2)	655(89.2)	<0.001
**Harmful to health**				
More than cigarettes	684 (66.8)	162 (57.4)	522 (70.4)	<0.001
Less than cigarettes	137 (13.3)	80 (27.8)	57 (7.8)	
Similar to cigarettes	211 (19.9)	43 (14.9)	168 (21.8)	
**Social acceptability is**				
More than cigarettes	332(33.3)	101 (35.2)	231 (32.6)	0.002
Less than cigarettes	345 (34.6)	111 (40.2)	234 (32.4)	
Similar to cigarettes	315 (32.1)	69 (24.7)	246 (35.1)	
**Waterpipe smoking is cool**	222 (29.2)	181 (70.7)	41 (8.3)	<0.001
**Females are more comfortable smoking waterpipe than cigarettes**	358 (37.0)	161 (57.8)	197 (28.8)	<0.001
**Waterpipe smoking makes males more attractive**	290 (28.1)	112 (39.1)	178 (23.9)	<0.001
**Waterpipe smoking makes females more attractive**	117 (11.6)	63 (22.3)	54 (7.6)	<0.001
**Waterpipe smokers have more friends**	446 (44.1)	176 (63.0)	27 (37.0)	<0.001
**Waterpipe smoking is part of our culture**	140 (13.8)	52 (19.3)	88 (11.7)	<0.001

Table 4 compares males and females in relation to these attitudes and beliefs about WPS. The findings indicate that there were statistically significant differences between males and females where males showed more “favorable” attitudes towards WPS. For example, 75% of males vs. 88% of females believed WPS is addictive.

**Table 4.** Comparison of all males and females in relation to knowledge and attitudes about WPS

	**Males (n=496)**	**Females (n=565)**	**P value (Design-based)**

	**Number (%)**	**Number (%)**	
**Waterpipe smoking may causes addiction**	350 (74.5)	482 (87.9)	<0.001
**Harmful to health**			
More than cigarettes	327(68.8)	357 (65.0)	
Less than cigarettes	88 (18.4)	49 (8.8)	<0.001
Similar to cigarettes	61 (12.7)	150 (26.2)	
**Social acceptability is**			
More than cigarettes	161 (34.5)	171 (32.2)	
Less than cigarettes	180 (38.4)	165 (31.2)	0.002
Similar to cigarettes	125 (27.1)	190 (36.6)	
**Waterpipe smoking is cool**	160 (41.8)	62 (16.5)	<0.001
**Females are more comfortable smoking waterpipe than cigarettes**	221 (49.4)	137 (26.3)	<0.001
**Waterpipe smoking makes males more attractive**	290 (30.9)	142 (25.7)	0.05
**Waterpipe smoking makes females more attractive**	77 (16.6)	40 (7.4)	<0.001
**Waterpipe smokers have more friends**	237 (50.7)	209 (38.4)	<0.001
**Waterpipe smoking is part of our culture**	56 (12.3)	84 (15.0)	0.18

### Factors associated with waterpipe smoking

Table 5 shows factors associated with WPS at the univariate level. Male gender, older age, city residence, smoking cigarettes, and alcohol use were all statistically significant factors associated with waterpipe smoking at the univariate level.

**Table 5.** Association between waterpipe smoking and potential risk factors

**Risk factors**	**All**	**Waterpipe Smoker**	**Non-waterpipe smoker**	**P value (Design-based)**

	**Number (%)**	**Number (%)**	**Number (%)**	
**Sex**				
Male	496 (100)	245 (48.9)	251 (51.1)	<0.001
Female	565 (100)	53 (9.5)	512 (90.5)	
**Age**				
18–20 years	373 (100)	82 (22.0)	291 (78.0)	<0.001
21–22 years	407 (100)	119 (29.1)	288 (70.9)	
23 year and over	281 (100)	97 (34.4)	184 (65.6)	
**Enrolment**				
College	244 (100)	66 (27.7)	178 (72.3)	0.90
Institute	817 (100)	232 (28.1)	585 (71.9)	
**Residence**				
Sulaimaniyah city	372 (100)	133 (35.9)	239 (64.1)	<0.001
Outside the city	664 (100)	161 (24.0)	503 (76.0))	
**Ethnicity**				
Kurdish	1051(100)	293 (27.8)	758 (72.2)	0.18
Other ethnic groups	8(100)	4(47.6)	4 (52.4)	
**Occasional cigarette smoker**				
Yes	217 (100)	161 (73.8)	56 (26.2)	<0.001
No	797 (100)	121 (15.2)	674 (84.8)	
**Regular cigarette smoker**				
Yes	100 (100)	80 (80)	20 (20)	<0.001
No	913 (100)	204 (22.3)	709 (77.7)	
**Parental cigarette smoking**				
Yes	230 (100)	71 (29.9)	159 (70.1)	0.36
No	801(100)	216 (27.1)	585 (72.9)	
**Alcohol and substance use**				
Yes	74 (100)	53 (69.9)	21 (30.1)	<0.001
No	943 (100)	229 (24.2)	714 (75.8)	

Factors that were significant at 0.2 or less were included in a multivariate logistic regression. Table 6 shows adjusted odds ratios or factors that remained significant at the multivariate level when a multiple logistic regression model was used. The statistically significant factors were male gender, ethnicity, residence in Sulaimani city, smoking cigarettes, and alcohol use. Compared to students not smoking cigarettes, cigarette smokers had 10-fold odds of being waterpipe smokers. Males had 5.7 times the odds of being waterpipe smokers compared to females. Similarly, residence in Sulaimani city, ethnicity, and consumption of alcohol were also independently significant risk factors for WPS (see Table 6).

**Table 6.** Adjusted odds ratios for factors significantly associated with waterpipe smoking

**Risk factor**	**Odds ratio (95% CI)**	***t***	***P value***
**Sex**			
Female	Reference group	9.3	*<0.001*
Male	5.68 (3.93–8.2)		
**Residence**			
Outside Sulaimaniyah	Reference group	2.2	*0.03*
Sulaimaniyah city	1.47 (1.04–2.07)		
**Ethnicity**			
Kurdish	Reference group	2.7	*0.006*
Other ethnicities	2.95 (1.58–15.89)		
**Cigarette Smoking**			
No	Reference group	12.0	*<0.001*
Yes	10.26 (7.02–15.01)		
**Alcohol/Substance use**			
No	Reference group	*2.8*	*0.004*
*Yes*	*2.79 (1.4–5.6)*		

Number of observations 967, F (4,951), P <0.001

## DISCUSSION

Consistent with the high prevalence of WPS we found in our study, a global review by Maziak et al. concluded that waterpipe smoking has become a global public health problem (14). The authors contributed the unexpected increase of WPS in the past 10 years to the introduction of flavored tobacco and the coffee shop culture, and its interaction with the social aspects of waterpipe smoking as well as the internet socialization facilities (14). Although there are no documented prevalence rates of waterpipe smoking in Iraqi Kurdistan, the current study clearly confirms a high prevalence (28%) of WPS similar to what has been reported in neighboring countries. For example, a study from Iran(6) reported a prevalence rate of 51% among university students. However, in our study the prevalence in females was significantly lower than in males, whereas in the Iranian study the rates were similar (males 52%, females 48%). This could be a true difference, but it could also be partly due to underreporting by females in our study due to the more conservative nature of the Kurdish society. Most other studies have reported lower overall prevalence rates and a preponderance of males compared to females. Results similar to our study have been reported from neighboring Arab countries. For example, a study from Jordan reported a prevalence rate of 30% (males 59%, females 13%)(8). Another study from Lebanon reported a similar prevalence rate of 30%(7), and a study from Pakistan reported a 19% prevalence rate (males 35%, females 13%) (9). One of earliest studies on waterpipe smoking in 2004 in Syria (10) reported a lower prevalence of 26% in males and 5% in females, which is an indication of the rising trend of WPS in the Middle East. Within the past ten years, Iraq has opened up to the world, the economic situation has improved, and more people have been traveling to neighboring countries. These factors have probably contributed to the rising prevalence of WPS in the country.

One of the alarming findings of the study is that 76% of waterpipe smokers had initiated WPS before joining the university. This indicates a high prevalence in adolescents and teenagers. Similar results have been reported from other countries such as Iran(5), Saudi Arabia(15), and Oman(3). Other findings of concern that could increase health risk and should be considered in any intervention were that one in five waterpipe smokers smoke on a daily basis and the vast majority of them share the mouthpiece.

With regard to attitudes towards WPS, one third of the participants believed WPS was more socially acceptable than cigarettes, and one in eight believed WPS was less harmful than cigarette smoking. With the high prevalence of WPS and these attitudes, non-waterpipe smoking students might be under more peer pressure to take up the behavior. These factors have to be considered in public health interventions addressing WPS. Similar attitudes and beliefs were reported in studies from Saudi Arabia(15), Bahrain(16), Syria(17), and Iran(18). The prevalence of these attitudes differed significantly between waterpipe smokers and non-smokers (Table 3) indicating the role of these factors in the spread of WPS among adolescents and the youth.

The independent risk factors of WPS were being a cigarette smoker (OR 10.3), male gender (OR 5.7), non-Kurdish ethnicity (OR 3.0), being a resident of the city (OR 1.5), and alcohol/substance use (OR 2.8). A Syrian study (10) reported similar findings including an OR of 3.8 for male gender, 4.0 for cigarette smoking, and 1.7 for city residence. Regular smoking, male gender, and positive attitudes were also reported as risk factors for hookah smoking in Iran(5). Availability of the facilities such as abundance of coffee shops with WPS facilities in the major cities and more socialization opportunities for males could explain why WPS is more common in males and in major cities. People who smoke cigarettes could find it easier to initiate WPS, which may explain why WPS is much more common in cigarette smokers. The effect of being a cigarette smoker on WPS was reported in a study from Jordan in which the odds ratio for males was 7.4 and 11.5 for females (19), which is comparable to our findings. Other risk factors for WPS reported in the literature include higher socioeconomic status and better parental education (14). However, these factors were not included in the present study.

The study has some strengths and limitations. This is the first study undertaken in Iraqi Kurdistan to investigate prevalence and associated factors of WPS. Although the study was conducted at only one university, it is one of the biggest universities in terms of the number of students and spread of campuses throughout the governorate. Nonetheless, generalizing the findings to all university students in the region should be done with caution. Like other cross-sectional surveys, information bias could not be ruled out. Making the questionnaire anonymous and informing participants on respecting privacy and confidentiality might have helped in reducing information bias.

## CONCLUSION

The present study confirms that WPS is highly prevalent among university students in Iraqi Kurdistan especially in males and it is becoming a public health problem. The study also showed prevalence of certain misconceptions and favorable attitudes of the youth about WPS that could be related to the spread of this method of tobacco use in the population. However, understanding this association requires further research. Tobacco control interventions should be designed by health authorities and their partners to address WPS among adolescents and the youth. Further research is required on prevalence and other aspects of WPS including high-school students in order to provide a better understanding of the problem and necessary data for planning socially responsive interventions.

## References

[1] World Health Organization WHO report on the global tobacco epidemic 2015: raising taxes on tobacco. World Health Organization; 2015.

[2] Maziak W Jawad M Jawad S Ward KD Eissenberg T Asfar T Interventions for waterpipe smoking cessation. Cochrane Database Syst Rev 2015 31;(7):CD005549. 10.1002/14651858.CD005549.pub3 PMC4838024 26228266

[3] Al-Lawati JA Muula AS Hilmi SA Rudatsikira E Prevalence and Determinants of Waterpipe Tobacco Use among Adolescents in Oman. Sultan Qaboos Univ Med J 2008;8(1):37–43. 21654955 PMC3087736

[4] Almerie MQ Matar HE Salam M Morad A Abdulaal M Koudsi A Maziak W Cigarettes and waterpipe smoking among medical students in Syria: a cross-sectional study. Int J Tuberc Lung Dis 2008;12(9):1085–91. 18713509 PMC2553239

[5] Fakhari A Mohammadpoorasl A Nedjat S Sharif Hosseini M Fotouhi A Hookah smoking in high school students and its determinants in Iran: a longitudinal study. Am J Mens Health 2015;9(3):186–92. 24855098 10.1177/1557988314535236

[6] Ghafouri N Hirsch JD Heydari G Morello CM Kuo GM Singh RF Waterpipe smoking among health sciences university students in Iran: perceptions, practices and patterns of use. BMC Res Notes 2011;4:496. 22087840 10.1186/1756-0500-4-496 PMC3279519

[7] Jradi H Wewers ME Pirie PR Binkley PF Ferketich K Cigarette and waterpipe smoking associated knowledge and behaviour among medical students in Lebanon. East Mediterr Health J 2013;19(10):861–8. 24313150

[8] Khabour OF Alzoubi KH Eissenberg T Mehrotra P Azab M Carroll MV Waterpipe tobacco and cigarette smoking among university students in Jordan. Int J Tuberc Lung Dis 2012;16(7):986–92. 22525279 10.5588/ijtld.11.0764 PMC3570564

[9] Haroon M Munir A Mahmud W Hyder O Knowledge, attitude, and practice of water-pipe smoking among medical students in Rawalpindi, Pakistan. J Pak Med Assoc 2014;64(2):155–8. 24640803

[10] Maziak W Fouad FM Asfar T Hammal F Bachir EM Rastam S Eissenberg T Ward KD Prevalence and characteristics of narghile smoking among university students in Syria. Int J Tuberc Lung Dis 2004;8(7):882–9. 15260281

[11] Lauritsen J Bruus M A comprehensive tool for validated entry and documentation of data Odense: EpiData Association.

[12] StataCorp 1985–2013 Stata Statistical Software. College Station, TX, USA: StataCorp LP.

[13] The Strengthening the Reporting of Observational Studies in Epidemiology 2009 [cited 2016 12 Jul]; Available from: http://www.strobe-statement.org/index.php?id=available-checklists.

[14] Maziak W Taleb ZB Bahelah R Islam F Jaber R Auf R The global epidemiology of waterpipe smoking. Tob Control 2015;24 Suppl 1:i3–i12. 25298368 10.1136/tobaccocontrol-2014-051903 PMC4345835

[15] Amin TT Amr MA Zaza BO Kaliyadan F Predictors of waterpipe smoking among secondary school adolescents in Al Hassa, Saudi Arabia. Int J Behav Med 2012;19(3):324–35. 21643931 10.1007/s12529-011-9169-2

[16] Borgan SM Marhoon ZA Whitford DL Beliefs and perceptions toward quitting waterpipe smoking among cafe waterpipe tobacco smokers in Bahrain. Nicotine Tob Res 2013;15(11):1816–21. 23674839 10.1093/ntr/ntt064

[17] Hammal F Mock J Ward KD Eissenberg T Maziak W A pleasure among friends: how narghile (waterpipe) smoking differs from cigarette smoking in Syria. Tob Control 2008;17(2):e3. 18375726 10.1136/tc.2007.020529

[18] Sabahy AR Divsalar K Nakhaee N Attitude of University Students towards Waterpipe Smoking: Study in Iran. Addict Health 2011;3(1–2):9–14. 24494111 PMC3905517

[19] McKelvey KL Wilcox ML Madhivanan P Mzayek F Khader YS Maziak W Time trends of cigarette and waterpipe smoking among a cohort of school children in Irbid, Jordan, 2008–11. Eur J Public Health 2013;23(5):862–7. 24078649 10.1093/eurpub/ckt140 PMC3784799

